# The effects of moderate neuromuscular blockade combined with transverse abdominal plane block on surgical space conditions during laparoscopic colorectal surgery: a randomized clinical study

**DOI:** 10.1186/s12871-022-01623-7

**Published:** 2022-04-04

**Authors:** Fang Ke, Zijin Shen, Cheng Wu, Lin Zhang, Rong Dong

**Affiliations:** 1 Department of Anesthesiology, Ruijin Hospital affiliated to Shanghai Jiaotong University School of Medicine, Shanghai, 200025 P.R. China; 2 Department of Health Statistics, Naval Medical University, Shanghai, 200433 China

**Keywords:** Transverse abdominal plane block (TAPB), Neuromuscular blockade monitoring, Surgical space conditions

## Abstract

**Background:**

Deep neuromuscular blockade may be beneficial on surgical space conditions during laparoscopic surgery. The effects of moderate neuromuscular blockade combined with transverse abdominal plane block (TAPB) on surgical space conditions during laparoscopic surgery have not been described. This work investigated whether the above combination is associated with similar surgical space conditions to those of deep neuromuscular blockade.

**Methods:**

Eighty patients undergoing elective laparoscopic surgery for colorectal cancer were randomly divided into two groups. The intervention group was treated with moderate neuromuscular blockade (train-of-four (TOF) count between 1 and 3) combined with TAPB (M group), while the control group was treated with deep neuromuscular blockade (D group), with a TOF count of 0 and a post-tetanic count (PTC) ≥1. Both groups received the same anesthesia management. The distance between the sacral promontory and the umbilical skin during the operation was compared between the two groups. The surgeon scored the surgical space conditions according to a five-point ordinal scale. Patients’ pain scores were evaluated 8 h after the operation.

**Results:**

The distance from the sacral promontory to the umbilical skin after pneumoperitoneum was similar between the D group and M group (16.03 ± 2.17 cm versus 16.37 ± 2.78 cm; *P =* 0.544). The 95% confidence intervals of the difference in the distance from the sacral promontory to the umbilical skin between the two groups were − 1.45–0.77 cm. According to the preset non-inferior standard of 1.5 cm, (− 1.45, ∞) completely fell within (− 1.50, ∞), and the non-inferior effect test was qualified. No significant difference was found in the surgical rating score between the two groups. The dosage of rocuronium in the group D was significantly higher than that in the group M (*P* < 0.01). The M group had significantly lower pain scores than the D group 8 h after the operation (*P* < 0.05).

**Conclusions:**

Moderate neuromuscular blockade combined with TAPB applied to laparoscopic colorectal cancer surgery can provide surgical space conditions similar to those of deep neuromuscular blockade. In addition, it reduces the use of muscle relaxants, relieves postoperative pain within 4 h after operation, and shorten the extubation time and stay in PACU when neostigmine was used as muscle relaxant antagonist.

**Trial registration:**

chictr.org.cn (ChiCTR2000034621), registered on July 12, 2020.

## Background

During the early years of laparoscopic surgery development, most anesthesiologists usually applied a moderate neuromuscular blockade due to the minimally invasive incision [1, 2]. A long intra-abdominal insufflation with moderate neuromuscular blockade leads to a decreased lung compliance and reduced functional residual capacity, leading to atelectasis. Carbon dioxide accumulation during a long intra-abdominal insufflation can cause hypercapnia and acidosis, resulting in insufficient perfusion of the abdominal organs and hemodynamic fluctuations [3, 4].

In recent years, anesthesiologists have applied deep neuromuscular blockade in laparoscopic surgery to improve surgical space conditions [5–7] so as to reduce the postoperative pain and shorten the recovery time [8–10]. However, deep neuromuscular blockade may increase the risk of residual neuromuscular blockade after the operation, leading to respiratory complications [11–13].

Local anesthetics can affect neuromuscular junction conduction through complex presynaptic and postsynaptic effects, thus enhancing the effect of non-depolarizing muscle relaxants [14, 15]. To our knowledge, no studies have investigated and published the impact of moderate neuromuscular blockade with transverse abdominal plane block (TAPB) on surgical space conditions during laparoscopic surgery. Our pilot study revealed that moderate neuromuscular blockade with TAPB could provide better surgical space conditions than moderate neuromuscular blockade alone.

This study was designed to assess the effects of moderate neuromuscular blockade with TAPB compared to deep neuromuscular blockade on surgical space conditions during laparoscopic surgery for colorectal cancer. Our hypothesis is that moderate neuromuscular blockade with TAPB might be non-inferior to deep blockade in providing the “optimal” surgical space conditions with potential benefits, as assessed by the surgeon .

## Methods

This study was performed in line with the principles of the Declaration of Helsinki and its later amendments or comparable ethical standards. The study was approved by the ethics committee of the Ruijin Hospital affiliated to the Shanghai Jiaotong University School of Medicine (No. 2020–007-1) and written informed consent was obtained from all subjects participating in the trial. The study was registered at chictr.org.cn prior to patient enrollment (ChiCTR2000034621, Principal investigator: Fang Ke, Date of registration: July 12, 2020).

In this study, patients undergoing surgery for colorectal cancer were enrolled on an elective date from August 2020 to February 2021. Patients had a grade I-III physical status classification according to the American Society of Anesthesiologists (ASA), were between 18 and 80 years of age, and had a body mass index (BMI) between 18 and 26 kg/m^2^. Patients suffering from neuromuscular junction diseases, severe heart, lung, liver, or kidney insufficiency, severe blood system disease, coagulation dysfunction, thrombocytopenia, or hemophilia were excluded from the study.

Participants were randomly divided into two groups: the deep neuromuscular blockade group (D Group) and the moderate neuromuscular blockade combined with TAPB group (M Group). A random number table method was adopted using Excel to allocate participants to the groups, and the grouping results were only known by the anesthesiologist. The patient’s blood pressure, electrocardiogram, pulse oxygen saturation, and anesthetic depth were routinely monitored throughout the entire operation. The depth of anesthesia was measured by Narcotrend (Monitor Technik, Bad Bramstedt, Germany). Oxygen was inhaled through a mask, and peripheral veins were catheterized for fluid administration.

A neuromuscular blockade monitor (E-NMT, GE Company, Finland) was used to stimulate the ulnar nerve and observe the contraction reaction of the adductor pollicis muscle. TOF counting with a frequency of 2 Hz, current of 60 mA, at 20-s intervals was adopted. PTC was monitored in the D Group every 10 min.

Propofol (2 mg/kg) and sufentanil (0.3 μg/kg) were administered to induce anesthesia. TOF count monitoring was performed after the patient lost consciousness, ensuring the basic values of 90–110% for three consecutive times. The D Group was treated with rocuronium 0.6 mg/kg (Esmeron®, N.V. Organon, Netherlands) through intravenous injection, while the M Group was intravenously treated with rocuronium 0.4 mg/kg. Continuous monitoring of neuromuscular blockade was conducted, and endotracheal intubation was performed when the TOF count was 0.

After endotracheal intubation, the researchers applied TAPB according to the patient’s group. The M Group received ultrasound-guided bilateral TAPB using ultrasound (Edge, Sonosite®, Fuji Film, Japan) and in-plane techniques. The patients lay supine, exposing the abdominal area between the costal margin and iliac crest. The ultrasound probe was placed transversely between the costal margin and the iliac crest, near the front or middle axillary line. Three layers of abdominal muscles were identified, including the external oblique muscle, the internal oblique muscle, and the transverse abdominal muscle. After the needle (Stimuplex® D, 0.71*120 mm 22 G*, B Braun Melsungen AG, Germany) was inserted between the internal oblique and transverse abdominal muscles, it was withdrawn to ensure that the needle tip was not in a blood vessel. Then, the local anesthetic (20 mL of 0.375% ropivacaine, Naropin®, AstraZeneca) was injected into each side.

The patients were placed in Trendelenburg position within 10 min of TAPB administration, the surgeon entered the room and prepped and draped the patient. Pneumoperitoneum was established 5 min after the beginning of the operation. Then, the surgeon (observer) measured the distance from the sacral promontory to the umbilical skin, while pneumoperitoneum pressure was controlled at 12 mmHg [5, 16]. During the operation, the surgeon scored the surgical space conditions according to a five-point ordinal scale ranging from 1 (extremely poor conditions) to 5 (optimal conditions) (Table 1) [17]. The data were recorded by the anesthesiologist. The surgeon didn’t know the group allocation.

**Table 1:** The surgical rating score for laparoscopic surgery

1	Extremely poor conditions: the surgeon is unable to work because of coughing or because of the inability to obtain a visible laparoscopic field because of inadequate muscle relaxation. Additional neuromuscular blocking agents must be given
2	Poor conditions: there is a visible laparoscopic field, but the surgeon is severely hampered by inadequate muscle relaxation with continuous muscle contractions, movements, or both with the hazard of tissue damage. Additional neuromuscular blocking agents must be given
3	Acceptable conditions: there is a wide visible laparoscopic field but muscle contractions, movements, or both occur regularly causing some interference with the surgeon’s work. There is the need for additional neuromuscular blocking agents to prevent deterioration
4	Good conditions: there is a wide laparoscopic working field with sporadic muscle contractions, movements, or both. There is no immediate need for additional neuromuscular blocking agents unless there is the fear of deterioration
5	Optimal conditions: there is a wide visible laparoscopic working field without any movement or contractions. There is no need for additional neuromuscular blocking agents

**Fig. 1:**
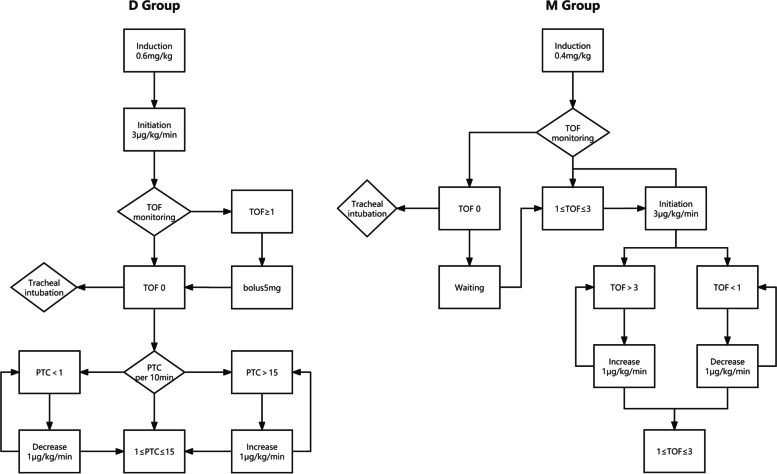
Flow chart of rocuronium management during surgery. TOF = train-of-four; PTC = post-tetanic count

Anesthesia was maintained by target control infusion (TCI) and desflurane. The TCI (Perfusor® Space Infusion Pump, B. Braun Melsungen AG.) effect compartment concentration (Marsh model) of propofol was 1 μg/mL, and remifentanil was sustainedly infused at 0.1 μg/kg/min. Desflurane (6%) was inhaled continuously, and the minimum alveolar concentration (MAC) was maintained between 0.7 and 1.0. The depth of anesthesia was maintained in the Narcotrend range of 30–50. A single dose of sufentanil (0.2 μg/kg) was administered before incision. Neuromuscular blockade was monitored and maintained by continuously infusing rocuronium (initial rate of 3 μg/kg/min). The TOF count in the D Group was maintained at 0 with PTC ≥ 1 [18]. The PTC was measured every 10 min during the operation. When PTC was > 15, the infusing rate of rocuronium was increased, and when PTC was < 1, the infusing rate was decreased. If TOF count occurred during the operation, a single dose of rocuronium (5 mg) was administered. The TOF count in the M Group was maintained within 1–3 [18]. The rocuronium infusing rate was increased when TOF count was 3 and decreased when TOF count was 1 (Fig. 1). When systolic blood pressure decreased by > 30% or mean arterial pressure (MAP) <65 mmHg for more than 3 min, phenylephrine (20 μg) or ephedrine (5 mg) was injected intravenously once.

The infusion of muscle relaxants ceased and a single dose of sufentanil (0.3 μg/kg) was administered 10 min before the end of the operation. Then, the patients were transferred to the post-anesthesia care unit as requested by the standard procedure in our institution. In this study, all patients were routinely subjected to antagonist treatment. When the TOF count recovered to 0.1, neostigmine (40 μg/kg, Xinyi, China) and atropine (20 μg/kg, Xinyi, China) were administered to antagonize the residual effects of the muscle relaxants. Coughing and swallowing reflexes were recovered when the patient regained consciousness. The endotracheal tube was removed after TOF ratio >0.9, establishing a steady breathing frequency of 10–20 breathes per minute and PetCO_2_ was ≤45 mmHg. Extubation time, total time in PACU and other adverse events (apnea, desaturation) were recorded.

Oxycodone was used for patient-controlled-analgesia (PCA); the background dose was 1 mg/h, the bolus dose was 1 mg/time, PCA was locked for 30 min after a bolus. The postoperative visual analogue scale (VAS) and bolus time follow-up were performed by a specialized anesthetic nurse, who did not know the group allocation.

### Outcome measures

The primary outcome measure was the distance from the sacral promontory to the umbilical skin and the surgeon’s subjective score of the surgical space conditions. Secondary outcome measures included the dosage of various narcotic drugs, the patient’s hemodynamic parameters during different periods of the operation, and patient pain scores (VAS) after the operation.

### Statistical analysis

Statistical analysis was performed using SPSS 20.0 software. The purpose of this clinical trial was to verify that the surgical space conditions of the M Group were not inferior to those in the D Group. Therefore, the sample size estimation formula for a non-inferior, parallel (1:1) clinical trial was: N_c_ = (Z_1-α_ + Z_1-β_) σ^2^(1 + 1/K)/(μ_T_-μ_C_ + ∆)^2^. According to the results of our pilot study, the mean μ_T_ value of the distance from the sacral promontory to the umbilical skin in the M Group was 16.03 cm, and the mean μ_C_ value in the D Group was 15.66 cm. The non-inferiority limit was set as ∆ = 1.5 cm. Assuming that the standard deviation (σ) was the same for both groups at 2.4, and α = 0.025, β = 0.10, and K = 1, then according to the formula, n_c_ = 35. Considering a drop-out rate of 10% in each group, the number of cases in each group should be no less than 39. Thus, 40 cases were included in each of the two groups in this study, which fulfills the requirements of the statistical tests.

Measurement data were expressed as mean ± standard deviation (^−^x ± s). Categorical data were expressed as numbers and percentages (%). The measurement data between two groups were compared by Student *t*-test; the categorical data were compared by Chi-square test (or Fisher’s exact test if a count less than five was expected), and Wilcoxon rank-sum test was used to compare ranked data. Bilateral 95% confidence intervals were calculated on the difference in surgical space measurements between the groups. The potential inferiority of the moderate neuromuscular blockade combined with TAPB on surgical space measurements compared to that of deep neuromuscular blockade was evaluated according to the preset non-inferior effect limit of 1.5 cm. If the lower boundary of the 95% confidence interval for μ_T_ - μ_C_ did not cross − 1.5 cm, the noninferiority of the M group to the D group was established. A value of *P* < 0.05 was considered statistically significant.

## Results

**Fig. 2:**
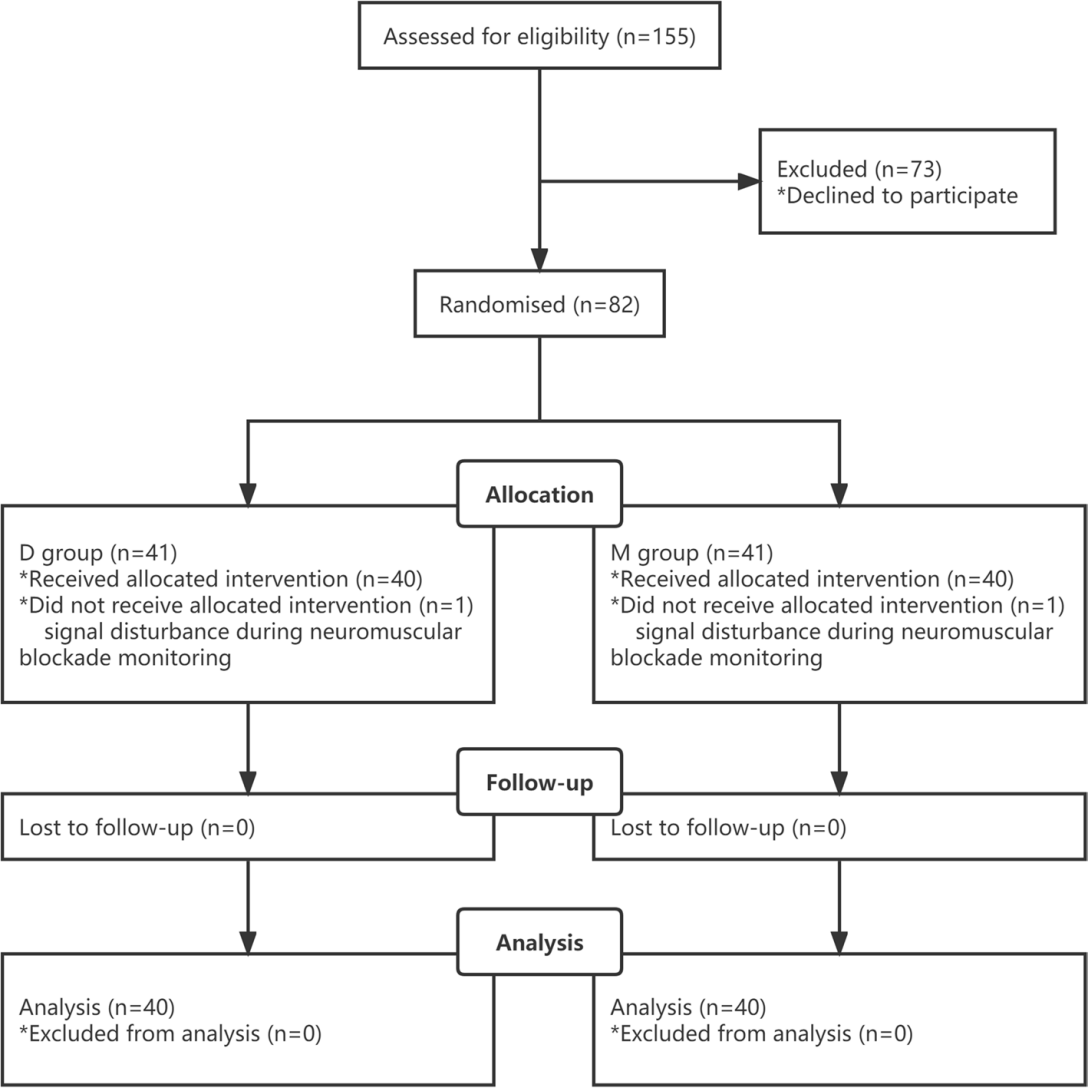
Consolidated Standards of Reporting Trials (CONSORT) flow diagram

Among the 155 patients selected for inclusion in the study, 73 refused to sign the informed consent and 82 participated in the study, but 2 patients were excluded due to signal disturbance during neuromuscular blockade monitoring. Eighty patients were finally included in the statistical analysis. Patients were randomly divided into the D (*n* = 40) and M (*n* = 40) Groups according to the different neuromuscular blockade management schemes (Fig. 2). Patient characteristics are listed in Table 2, which show that the two treatment groups were similar in physical characteristics, gender, preoperative examination, types of surgery, and hemodynamic variables.

**Table 2:** Baseline demographic and clinical characteristics of each group

	D Group (*n* = 40)	M Group (*n* = 40)	*P*
ASA (I/II/III)	8/28/4	11/27/2	0.309
Gender (male/female)	24/16	24/16	1.000
Age (years)	60 ± 1	60 ± 2	0.972
Height (cm)	163 ± 1.3	166 ± 1.1	0.049
Weight (kg)	63 ± 1.5	64 ± 1.5	0.490
BMI (kg/m^2^)	23.6 ± 0.47	23.4 ± 0.42	0.728
Total bilirubin (umol/L)	11.73 ± 0.68	13.75 ± 0.74	0.049
Direct bilirubin (umol/L)	2.28 ± 0.14	2.19 ± 0.13	0.665
Total protein (g/L)	67.85 ± 0.84	67.68 ± 0.74	0.876
Albumin (g/L)	39.2 ± 0.5	39 ± 0.5	0.786
Creatinine (umol/L)	78.1 ± 2.2	79.8 ± 2.3	0.586
Urea nitrogen (mmol/L)	5.23 ± 0.23	4.88 ± 0.2	0.263
Hemoglobin (g/L)	123 ± 3.4	129 ± 2.7	0.199
Hematocrit	0.43 ± 0.03	0.37 ± 0.01	0.390
Type of surgery (n)			0.107
Right/Left hemicolectomy	11	7	
Sigmoidectomy	1	6	
Rectum anterior resection	28	27	
Systolic blood pressure (mmHg)	139.9 ± 20.4	138.4 ± 22.4	0.755
Diastolic blood pressure (mmHg)	78.8 ± 11.7	79.3 ± 12.0	0.851
Heart rate (min^−1^)	71.2 ± 10.7	71.1 ± 11.7	0.976

*ASA* American Society of Anesthesiologists, *BMI* body mass index

The distance from the sacral promontory to the umbilical skin after pneumoperitoneum was similar between the D group and M group (16.03 ± 2.17 cm versus 16.37 ± 2.78 cm; *P* = 0.544). The 95% confidence interval of the difference in the distance between the M and D group was − 1.45 ~ 0.77 cm. The lower boundary of this 95% CI did not cross − 1.5 cm, and as such, the noninferiority of the M group to the D group was established.

No difference was observed in the surgical rating score of the surgical space conditions after the operation between the two groups. In the D Group, 34 patients had a score of 5 points, five patients had a score of 4 points, and one patient had a score of 3 points. In the M group, 35 patients scored 5 points, and five patients had a score of 4 points.

Except for the different schemes for neuromuscular blockade management, other drugs for maintaining anesthesia were the same in both groups. The rocuronium dosage in the D Group was significantly higher than that used in the M Group (83.6 ± 3.6 mg vs 69.2 ± 3.1 mg, *P* < 0.01). No significant difference was observed in the fluid balance, duration of anesthesia, operative time, pneumoperitoneum time and hemodynamic variables between the two groups (*P* > 0.05, Table 3).

**Table 3:** Comparison of primary and secondary outcomes between the two groups

	D Group (*n* = 40)	M Group (*n* = 40)	*P*
**Primary outcome measures**			
Surgical space measurement (cm)	16.03 ± 2.17	16.37 ± 2.78	
Surgical rating score (1/2/3/4/5 points)	0/0/1/5/34	0/0/0/5/35	0.717
**Secondary outcome measures**			
Rocuronium bromide (mg)	83.6 ± 3.6	69.2 ± 3.1	0.003
Remifentanil (mg)	0.99 ± 0.08	1.01 ± 0.08	0.435
Propofol (mg)	510 ± 35.7	524 ± 32.8	0.770
Sufentanil (μg)	46 ± 1.2	43 ± 1.9	0.202
Colloid (mL)	1081 ± 53.3	906 ± 86.6	0.089
Crystal (mL)	1791 ± 63.5	1605 ± 76.3	0.065
Urine volume (mL)	566 ± 75	535 ± 55	0.737
Blood loss (mL)	150 ± 20	145 ± 18	0.853
Operation time (min)	146.7 ± 7.7	141.9 ± 8.6	0.676
Anesthesia time (min)	171.3 ± 7.8	170.4 ± 9.2	0.939
Pneumoperitoneum time (min)	106.3 ± 6	103.2 ± 7	0.747
Systolic blood pressure (mmHg)			
Anesthesia induction	104.8 ± 12.5	105.6 ± 17.8	0.800
Operation for 1 h	107.1 ± 9.1	108.4 ± 11.7	0.595
Operation for 2 h	107.5 ± 10.0	103.4 ± 19.2	0.240
After operation	109.63 ± 10.2	108.8 ± 12.7	0.736
Diastolic blood pressure (mmHg)			
Anesthesia induction	62.6 ± 10.1	63.9 ± 8.8	0.526
Operation for 1 h	62.7 ± 6.3	66.2 ± 8.0	0.320
Operation for 2 h	61.6 ± 8.1	62.0 ± 6.6	0.799
After operation	61.4 ± 7.6	62.6 ± 7.9	0.481
Heart rate (min^−1^)			
Anesthesia induction	65.4 ± 9.4	63.0 ± 9.4	0.262
Operation for 1 h	65.1 ± 9.2	60.3 ± 5.6	0.08
Operation for 2 h	65.8 ± 9.1	63.2 ± 6.6	0.16
After operation	65.0 ± 8.1	64.0 ± 7.2	0.569
Extubation time (min)	16.83 ± 4.97	11.06 ± 4.33	0.001
Total time in PACU (min)	34.63 ± 5.42	26.48 ± 5.21	0.001
Apnea immediately after extubation (n)	2	1	1.000
SpO2 < 93% immediately after extubation	4	5	1.000
Apnea within 30 min of extubation	0	0	
SpO2 < 93% within 30 min of extubation	0	0	
**VAS**			
1 h post	2.73 ± 0.93	1.67 ± 0.94	0.001
4 h post	3.58 ± 0.71	2.73 ± 0.60	0.001
8 h post	4.10 ± 0.78	3.88 ± 0.88	0.230
Total bolus times within 24 h	10.40 ± 1.92	8.28 ± 1.72	0.001

*VAS* visual analogue scale

The VAS of the M Group was significantly lower at 1 (2.73 ± 0.93 vs 1.67 ± 0.94, *P* < 0.01) and 4 (3.58 ± 0.71 vs 2.73 ± 0.60, *P* < 0.01) hours after the operation. Total bolus times within 24 h (10.40 ± 1.92 vs 8.28 ± 1.72, *P* < 0.01) were less in the M Group. No significant difference in VAS was found between the two groups at 8 h after operation. Neostigmine and atropine were administered instead of sugammadex due to the high price of the latter, to antagonize the residual effects of the muscle relaxants when the TOF count recovered to 0.1. The extubation time (16.83 ± 4.97 min vs 11.06 ± 4.33 min, *P* < 0.01) and the total time in PACU (34.63 ± 5.42 min vs 26.48 ± 5.21 min, *P* < 0.01) was significantly less in the M Group.

Adverse events, including apnea and desaturation (SpO2 < 93%) immediately after extubation and within 30 min of extubation are summarized in Table 3. The differences were not statistically significant. All events of desaturation were resolved using the jaw-thrust maneuver and supplementary oxygen.

## Discussion

Our study revealed that in laparoscopic colorectal surgery, moderate neuromuscular blockade combined with TAPB provides surgical space conditions similar to those of deep neuromuscular blockade.

In recent years, a growing number of anesthesiologists have applied deep neuromuscular blockade technology in laparoscopic surgery to provide surgeons with better surgical space conditions so as to improve abdominal organ perfusion and alleviate postoperative pain for laparoscopic surgery [19–22]. The results of a meta-analysis by Bruintjes et al. demonstrate that deep neuromuscular blockade improves surgical space conditions compared with moderate neuromuscular blockade in laparoscopic surgery. This study quantified surgical space conditions by measuring the distance from the sacral promontory to the umbilical skin. The results indicate that deep neuromuscular blockade increased the distance from the sacral promontory to the umbilical skin [5]. Our study used this method to evaluate the surgical conditions during laparoscopic surgery. No significant difference in surgical space measurements was observed between the M Group and G Group.

A study on bariatric surgery published in PLOS One in 2016 included 100 obese patients. The researchers randomly divided all patients into moderate and deep neuromuscular blockade groups. Surgeons evaluated the surgical space conditions according to the operation evaluation scale. The results showed that the surgeon’s operation evaluation results were maintained at five points in the deep neuromuscular blockade group, and the patients suffered from less pain in the post-anesthesia care unit (*P* < 0.05), including less shoulder pain (*P* < 0.05) [16]. Our results showed that both moderate neuromuscular blockade combined with TAPB and deep neuromuscular blockade could provide excellent surgical space conditions.

Furthermore, a number of studies showed reduced muscle injury after deep neuromuscular blockade during total hip replacement and the levels of IL-6, CPK, and LDH were lower after the operation [23, 24]. Deep neuromuscular blockade can help to reduce muscle injury and reduce the stress of an operation and perioperative inflammation to a certain extent through improving surgical space conditions and making the operation easier to perform for the surgeon [25–27]. However, deep neuromuscular blockade is not that easy to popularize because of the prolonged recovery time and increased incidence of residual neuromuscular blockade after the operation [11]. Another possible reason can be that Sugammadex, the rocuronium specific antagonist, is quite expensive in China and out of the medical insurance list. Our study showed a prolonged extubation time and PACU residence time in the D Group. Kopman et al. proposed that the antagonism of neostigmine was slow and incomplete when using deep neuromuscular blockade, leading to an increase in the incidence of postoperative residual neuromuscular blockade [28].

A research showed that local anesthetics can enhance the effect of muscle relaxants caused by rocuronium [15]. Wang et al. found that the increased adult muscle-type nicotinic acetylcholine receptor inhibition produced when local anesthetics are combined with nondepolarizing muscle relaxants may contribute to the clinical enhancement of neuromuscular blockade by local anesthetics [14]. These studies may explain the reason why moderate neuromuscular blockade combined with TAPB can achieve similar effect of deep muscle relaxation in our study.

The application of deep neuromuscular blockade in laparoscopic surgery is recognized as the optimal choice (keeping the TOF count at 0 and PTC ≥ 1) [5]. Deep neuromuscular blockade combined with TAPB can provide good surgical space conditions and exert an appropriate analgesic effect compared with simple deep neuromuscular blockade [29]. Deep neuromuscular blockade combined with TAPB group was not included in this study. During our pilot study, three groups were investigated and compared, such as deep neuromuscular blockade, moderate neuromuscular blockade combined with TAPB, and moderate neuromuscular blockade alone. Surgeons complained of a poor neuromuscular blockage effect for five patients in the moderate neuromuscular blockade group; thus, additional neuromuscular blockade was required to complete the operation. Consequently, this study did not consider anymore the moderate neuromuscular blockade group.

In this study, the TAPB technique was guided by ultrasound, mastered in an accurate and safe operation by most anesthesiologists. In this study, TAPB slightly alleviated the pain within 4 h after operation, similar to what shown in previous studies [30, 31].

This study has several limitations. The sample size of this study was relatively small, and the detection of differences in some adverse events, such as apnea and desaturation was probably underpowered. The major side effects of neuromuscular blockade focused on postoperative respiratory function, including postoperative hypoxemia, airway obstruction, and muscle weakness, resulting in an increased risk of postoperative respiratory complications [12]. In addition, the length of hospital stay and ICU stay was not compared and long-term follow-up data from the patients were not collected.

## Conclusions

Moderate neuromuscular blockade combined with TAPB applied to laparoscopic colorectal cancer surgery can provide surgical space conditions similar to those of deep neuromuscular blockade. In addition, it reduces the use of muscle relaxants, relieves postoperative pain within 4 h after operation, shorten the extubation time and stay in PACU when neostigmine was used as muscle relaxant antagonist.

## Data Availability

The datasets used and/or analyzed during the current study are available from the corresponding author on reasonable request.

## Abbreviations

TAPB: Transverse abdominal plane block
TOF: Train-of-four
PTC: Post-tetanic count
VAS: Visual analogue scale
MAC: Minimum alveolar concentration
ASA: American Society of Anesthesiologists
BMI: Body mass index
TCI: Target control infusion
MAP: Mean arterial pressure
PCA: Patient-controlled analgesia

## References

[1] King M, Sujirattanawimol N, Danielson DR, Hall BA, Schroeder DR, Warner DO (2000). Requirements for muscle relaxants during radical retropubic prostatectomy. Anesthesiology.

[2] Bai Y, Ren H, Luo A, Huang Y, Ye T, Guo X (2010). Effects of residual paralysis after a single intubating dose of rocuronium on postoperative pulmonary function of patients undergoing laparoscopic gynecological surgeries. Acta Acad Med Sinicae.

[3] Cho YJ, Paik H, Jeong SY, Park JW, Jo WY, Jeon Y, Lee KH, Seo JH (2018). Lower intra-abdominal pressure has no cardiopulmonary benefits during laparoscopic colorectal surgery: a double-blind, randomized controlled trial. Surg Endosc.

[4] Diaz-Cambronero O, Flor Lorente B, Mazzinari G, Vila Montañes M, García Gregorio N, Robles Hernandez D, Olmedilla Arnal LE, Argente Navarro MP, Schultz MJ, Errando CL (2019). A multifaceted individualized pneumoperitoneum strategy for laparoscopic colorectal surgery: a multicenter observational feasibility study. Surg Endosc.

[5] Bruintjes MH, van Helden EV, Braat AE, Dahan A, Scheffer GJ, van Laarhoven CJ, Warlé MC (2017). Deep neuromuscular block to optimize surgical space conditions during laparoscopic surgery: a systematic review and meta-analysis. Br J Anaesth.

[6] Jacob R, Joseph HW, Manfred B, Mulier Jan P, Niels R, Tiffany W, Li Michael K, Peter G, Assaid Christopher A, Hein F, Armin S (2017). Deep neuromuscular blockade improves laparoscopic surgical conditions: a randomized, controlled Study. Adv Ther.

[7] Özdemir-van Brunschot DMD, Braat AE, van der Jagt MFP, Scheffer GJ, Martini CH, Langenhuijsen JF, Dam RE, Huurman VA, Lam D, d'Ancona FC, Dahan A, Warlé MC (2018). Deep neuromuscular blockade improves surgical conditions during low-pressure pneumoperitoneum laparoscopic donor nephrectomy. Surg Endosc.

[8] Bruintjes Moira HD, Piet K, Martini Chris H, Poyck Paul P, d'Ancona Frank CH, Huurman Volkert AL, van der Jagt M, Langenhuijsen JF, Nijboer Willemijn N, van Laarhoven Cornelis JHM, Albert D, Warlé Michiel C (2019). RELAX cg: efficacy of profound versus moderate neuromuscular blockade in enhancing postoperative recovery after laparoscopic donor nephrectomy: a randomised controlled trial. Eur J Anaesthesiol.

[9] Dubois Philippe E, Laurie P, Jacques J, Maria-Laura M, Maximilien G, Donnez O (2014). Deep neuromuscular block improves surgical conditions during laparoscopic hysterectomy: a randomised controlled trial. Eur J Anaesthesiol.

[10] Marjolijn L, Ulas BL, Apers Jan A, Erwin B, Verbrugge Serge J C, Dunkelgrun M. Low-pressure pneumoperitoneum with deep neuromuscular blockade in metabolic surgery to reduce postoperative pain: a randomized pilot trial. Surg Endosc. 2020;35:2838–45. 10.1007/s00464-020-07719-w 32556699

[11] Fuchs-Buder T, Nemes R, Schmartz D (2016). Residual neuromuscular blockade: management and impact on postoperative pulmonary outcome. Curr Opin Anaesthesiol.

[12] Kirmeier E, Eriksson LI, Lewald H, Jonsson Fagerlund M, Hoeft A, Hollmann M, Meistelman C, Hunter JM, Ulm K, Blobner M (2019). Post-anaesthesia pulmonary complications after use of muscle relaxants (POPULAR): a multicentre, prospective observational study. Lancet Respir Med.

[13] McLean Duncan J, Daniel D, Farhan Hassan N, Ladha Karim S, Tobias K, Matthias E (2015). Dose-dependent association between intermediate-acting neuromuscular-blocking agents and postoperative respiratory complications. Anesthesiology.

[14] Wang H, Zhang Y, Li ST (2010). The effect of local anesthetics on the inhibition of adult muscle-type nicotinic acetylcholine receptors by nondepolarizing muscle relaxants. Eur J Pharmacol.

[15] Carvalho VH, Braga Ade F, Braga FS, Loyola YC, de Araujo DR, Mantovani M (2009). The influence of lidocaine and racemic bupivacaine on neuromuscular blockade produced by rocuronium. A study in rat phrenic nerve-diaphragm preparation. Acta Cir Bras.

[16] Torensma B, Martini CH, Boon M, Olofsen E (2016). In 't veld B, Liem RS, Knook MT, swank DJ, Dahan a: deep neuromuscular block improves surgical conditions during bariatric surgery and reduces postoperative pain: a randomized double blind controlled trial. PLoS One.

[17] Martini CH, Boon M, Bevers RF, Aarts LP, Dahan A (2014). Evaluation of surgical conditions during laparoscopic surgery in patients with moderate vs deep neuromuscular block. Br J Anaesth.

[18] Naguib M, Brull SJ, Kopman AF, Hunter JM, Fülesdi B, Arkes HR, Elstein A, Todd MM, Johnson KB (2018). Consensus statement on perioperative use of neuromuscular monitoring. Anesth Analg.

[19] Loh PS, Yeong CH, Masohood NS, Sulaiman N, Zaki RA, Fabell K, Abdullah B (2021). Comparison of deep and moderate neuromuscular blockade in microwave ablation of liver tumours: a randomized-controlled clinical trial. Sci Rep.

[20] Zhu SJ, Zhang XL, Xie Q, Zhou YF, Wang KR (2020). Comparison of the effects of deep and moderate neuromuscular block on respiratory system compliance and surgical space conditions during robot-assisted laparoscopic radical prostatectomy: a randomized clinical study. J Zhejiang Univ Sci B.

[21] Zhang XF, Li DY, Wu JX, Jiang QL, Zhu HW, Xu MY (2018). Comparison of deep or moderate neuromuscular blockade for thoracoscopic lobectomy: a randomized controlled trial. BMC Anesthesiol.

[22] Staehr-Rye AK, Rasmussen LS, Rosenberg J, Juul P, Lindekaer AL, Riber C, Gätke MR (2014). Surgical space conditions during low-pressure laparoscopic cholecystectomy with deep versus moderate neuromuscular blockade: a randomized clinical study. Anesth Analg.

[23] Oh CS, Lim HY, Jeon HJ, Kim TH, Park HJ, Piao L, Kim SH (2021). Effect of deep neuromuscular blockade on serum cytokines and postoperative delirium in elderly patients undergoing total hip replacement: a prospective single-blind randomised controlled trial. Eur J Anaesthesiol.

[24] Eun KJ, Kee MS, Eunji H, Dongchul L, Yeop KJ, Kwak HJ (1935). Effects of deep neuromuscular block with low-pressure pneumoperitoneum on respiratory mechanics and biotrauma in a steep Trendelenburg position. Sci Rep.

[25] Mulier Jan P, Dillemans B (2019). Anaesthetic factors affecting outcome after bariatric surgery, a retrospective Levelled regression analysis. Obes Surg.

[26] Kyeong OS, Woo-Keun K, Sangwoo P, Gi JS, Han KJ, Youn-Kwan P, Young LS, Lim BG (2019). Comparison of operating conditions, postoperative pain and recovery, and overall satisfaction of surgeons with deep vs. no neuromuscular blockade for spinal surgery under general anesthesia: a prospective randomized controlled trial. J Clin Med.

[27] Koo CH, Chung SH, Kim BG, Min BH, Lee SC, Oh AY, Jeon YT, Ryu JH (2019). Comparison between the effects of deep and moderate neuromuscular blockade during transurethral resection of bladder tumor on endoscopic surgical condition and recovery profile: a prospective, randomized, and controlled trial. World J Urol.

[28] Kopman AF, Naguib M (2015). Laparoscopic surgery and muscle relaxants: is deep block helpful. Anesth Analg.

[29] Qi-Hong SXZ, Xu SYC, Ke LRW (2021). Comparison of ultrasound-guided erector Spinae plane block and oblique subcostal transverse Abdominis plane block for postoperative analgesia in elderly patients after laparoscopic colorectal surgery: a prospective randomized study. Pain Ther.

[30] Børglum J, Gögenür I, Bendtsen TF (2016). Abdominal wall blocks in adults. Curr Opin Anaesthesiol.

[31] Mirra A, von Rotz A, Schmidhalter M, Moser L, Casoni D, Spadavecchia C (2018). Ultrasound-guided lateral and subcostal transversus abdominis plane block in calves: a cadaveric study. Vet Anaesth Analg.

